# Preliminary Investigation of a Transcutaneous Ultrasound-Guided Technique for Pudendal Nerve Block in Six Horse Cadavers

**DOI:** 10.3390/ani16060995

**Published:** 2026-03-23

**Authors:** Elliot Pye, Miguel Gozalo Marcilla, Juliet C. Duncan

**Affiliations:** The Royal (Dick) School of Veterinary Studies, Easter Bush Campus, The University of Edinburgh, Roslin EH25 9RG, UK; miguel.marcilla@ed.ac.uk (M.G.M.); juliet.duncan@ed.ac.uk (J.C.D.)

**Keywords:** horse, pudendal nerve, regional anaesthesia, ultrasonography

## Abstract

Regional anaesthesia of the equine anogenital tract is limited to local infiltration, extradural, blind palpation and nerve stimulator-guided techniques which risk iatrogenic damage, recumbency and ataxia. This study aimed to describe and assess the feasibility of transcutaneous ultrasound-guided (USG) pudendal nerve staining in equine cadavers. An initial pilot phase used two fresh equine cadavers and one standing unsedated horse to image the intrapelvic anatomy using ultrasound. One fixed equine cadaver specimen was also dissected to identify the pudendal nerve and refine the dissection approach. The main study of six fresh equine cadavers used ultrasound to identify the landmarks of pelvic brim, pelvic urethra, rectum and semimembranosus musculature. Bilateral USG injection of 15 mL of methylene blue dye was performed, followed by anatomical dissection. The pudendal nerve was stained in 41.7% of injections, with an average staining length of 8.26 cm. Landmarks guiding dye injection were reliably imaged in all cadavers. Imaging of the pudendal nerve was not possible. Staining of the sciatic nerve did not occur. Transcutaneous USG pudendal nerve staining with methylene blue dye (15 mL) is possible in equine cadavers. However, this technique cannot be currently recommended. Further refinement in cadavers is necessary to improve the staining success rate.

## Simple Summary

Techniques for desensitising the area around the anus and genitals (anogenital) in horses are limited. This leaves veterinary surgeons with few proven options for local anaesthesia in horses undergoing anogenital surgery. Local anaesthetic has been used successfully in horses to block the two pudendal nerves that innervate the anogenital area, though current techniques carry risks such as damage to blood vessels and other organs. An ultrasound-guided (USG) technique for equine anogenital desensitisation may overcome these drawbacks. The aim of this study was to describe and assess the feasibility of using an USG technique, to identify the two pudendal nerves in horse cadavers and inject dye around them as a model of USG pudendal nerve blockade. After a pilot phase to determine the ultrasonographic and dissection approach, six fresh horse cadavers received USG injections of methylene blue dye (15 mL) to both pudendal nerves, guided by the landmarks identified in the pilot phase. Dissection revealed that the pudendal nerve was stained in 41.7% of injections. Based on these results, significant refinement of the technique in further cadaver studies is necessary to improve the staining success rate.

## 1. Introduction

Ultrasound-guided (USG) regional anaesthesia is a rapidly progressing area of veterinary anaesthesia. Ultrasound guidance has been shown to improve local block efficacy compared to the use of nerve-stimulator techniques in human patients [1]. Despite this, the development of USG techniques in equine anaesthesia is slowly progressing [2,3,4], with regional techniques being used in only 18% of all equine anaesthetic cases [5]. The underuse and scarcity of new research into USG local anaesthetic techniques in horses currently leaves equine clinicians with a limited number of evidence-based regional techniques [6].

Historically, systemic analgesics such as non-steroidal anti-inflammatory drugs (NSAIDs) [7] and opioids [8] have been the mainstay for providing analgesia in horses undergoing surgical procedures, in addition to basic local anaesthetic techniques [6]. Equine anogenital regional techniques include local infiltration of external tissues, blind techniques guided by manual palpation or a peripheral nerve stimulator and extradural techniques. However, all blind techniques have an inherent risk of iatrogenic damage, and the success rate relies heavily on user experience [9]. Additionally, extradural techniques in standing horses can produce recumbency and ataxia, or indeed complete block failure from inappropriate technique or anatomical abnormalities [10]. The use of ultrasound guidance for regional anaesthesia of the equine anogenital tract may overcome these drawbacks.

The equine pudendal nerve is a mixed motor and sensory nerve, originating from spinal nerves S2–4. Bilaterally paired pudendal nerves follow the medial surface of the sacrosciatic ligaments and branch into the caudal rectal nerve, the perineal nerve and the dorsal nerve of penis or clitoris [11]. The pudendal nerve and its branches provide innervation to the perineal musculature and skin, anus, vulva, udder, scrotum, prepuce, penis and clitoris [12]. Blind pudendal nerve blockade has been described in horses, guided by rectal palpation [13] and peripheral nerve stimulation [12]. Complications secondary to blind techniques involve rectal or vaginal puncture, vascular laceration, intraneural injection and intravascular injection of local anaesthetic [12]. The risk for iatrogenic harm may be reduced with the benefit of continuous ultrasound guidance.

Ultrasound-guided pudendal nerve blockade has been described in cats [14,15], donkeys [16], dogs [17,18] and sheep [19] and has been shown to provide superior post-operative analgesia compared to extradural techniques in man [20]. However, the development of an USG technique has not yet been described in horses.

The objectives of this study were: (i) to describe a transcutaneous USG technique for pudendal nerve staining in equine cadavers and (ii) to assess the feasibility of performing USG pudendal nerve staining in the horse. It was hypothesised that (i) the pudendal nerve and surrounding anatomy can be reliably imaged using ultrasound in equine cadavers and (ii) the target structures can be stained under ultrasound-guidance with methylene blue dye.

## 2. Materials and Methods

Ethical approval for this study was gained from The University of Edinburgh Veterinary Ethical Review Committee (reference 160.23). Informed written owner consent was obtained for all horses used in the study. When cadavers were used, euthanasia was performed for reasons unrelated to the study and owner consent was gained as part of the standard Dick Vet Equine Hospital euthanasia consent form. Our exclusion criteria included cadavers with abnormalities of the pelvic or perineal anatomy. The reporting of this study followed the CACTUS guidelines [21] (Supplementary File S1). Generative artificial intelligence was used only to enhance drawings and diagrams for illustrative purposes.

### 2.1. Pilot Study (Two Fresh Equine Cadavers, One Standing Horse and One Fixed Equine Cadaver)

Pilot imaging studies of two fresh equine cadavers were undertaken prior to data collection to identify potential difficulties with performing the imaging technique. The use of an ultrasound machine (Vivid iQ, GE Healthcare, Chalfont St Giles, UK) with both linear (12L-RS, GE Healthcare, Chalfont St Giles, UK) and curvilinear (C1-5-RS, GE Healthcare, Chalfont St Giles, UK) ultrasound probes, positioned ventrolaterally, laterally and dorsolaterally to the anus, was evaluated. Gain was manually adjusted at each position. The above enabled assessment of: optimal cadaver positioning; ultrasound probe type and position; ultrasound machine settings; the impact of body condition score (BCS) and perineal conformation on imaging; and finally, the feasibility of imaging intrapelvic structures with ultrasound.

Following this, a third pilot imaging study of an unsedated standing teaching horse was undertaken to compare ultrasonographic visualisation of the intrapelvic anatomy in the standing horse with that of the two recumbent cadaver specimens in the previous part of the pilot study. Colour flow Doppler was used to identify vasculature. The imaging data from these pilot studies were not included in the analysis of the main study.

Finally, dissection of another equine cadaver specimen (fixed in formaldehyde 5%) was undertaken to identify the anatomy of the pudendal nerve and optimise the dissection approach for the main study.

### 2.2. Main Study (Six Fresh Equine Cadavers)

All eligible cadavers that presented to the clinic over a period of 6 months from December 2023 to May 2024 were used. The process described below started 8 weeks after the pilot phase. All imaging, injection and dissection was performed by the same researcher E.P, a second to third-year resident of the European College of Veterinary Anaesthesia and Analgesia (ECVAA) with 5 years of experience in USG regional techniques in large and small animal clinical cases and experimental animal studies.

Before injection, cadavers were positioned in dorsal or lateral recumbency depending on the location of euthanasia and the ability to alter the cadaver position. The perineal skin was cleared of gross debris and long hair was clipped. The final skin preparation was performed with alcohol. Ultrasound gel was applied to the skin and to the ultrasound probe.

**Figure 1:**
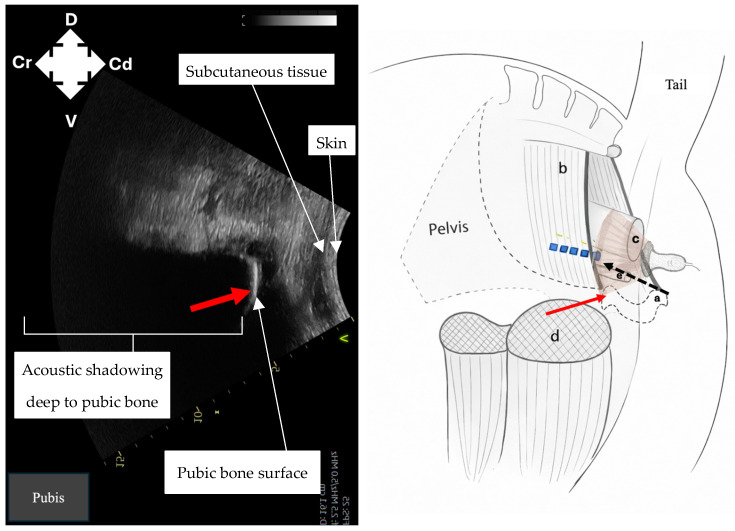
Ultrasound image and corresponding ultrasound probe placement to identify the pubis (red arrow) to guide needle placement (black dashed line) for pudendal nerve (blue dashed line) staining in equine cadavers using the described ultrasound technique. a: pubis; b: sacrosciatic ligament; c: rectum; d: semimembranosus muscle; e: pelvic urethra; D: dorsal; V: ventral; Cr: cranial; Cd: caudal.

**Figure 2:**
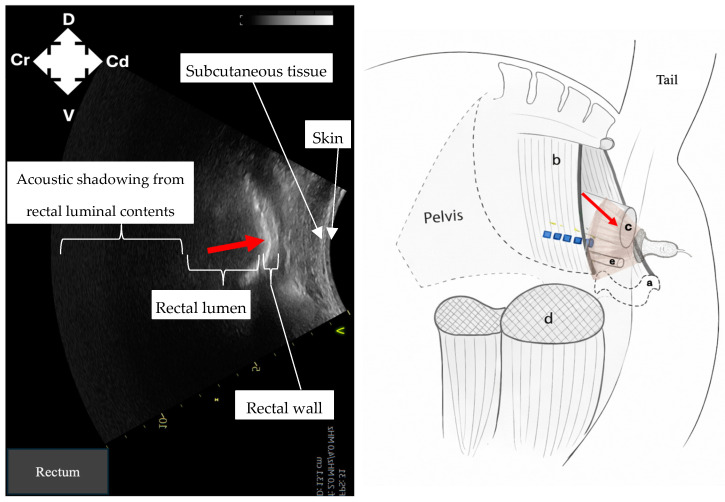
Ultrasound image and corresponding ultrasound probe placement to identify the rectum (red arrow) to guide needle placement for pudendal nerve (blue dashed line) staining in equine cadavers using the described ultrasound technique. a: pubis; b: sacrosciatic ligament; c: rectum; d: semimembranosus muscle; e: pelvic urethra; D: dorsal; V: ventral; Cr: cranial; Cd: caudal.

**Figure 3:**
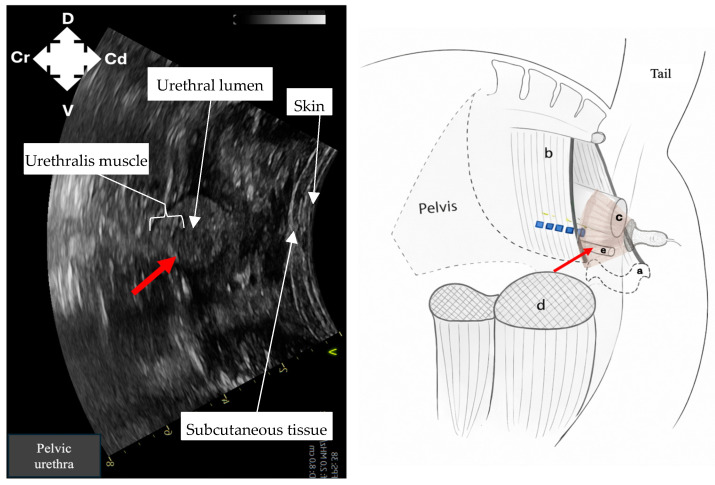
Ultrasound image and corresponding ultrasound probe placement to identify the pelvic urethra (red arrow) to guide needle placement for pudendal nerve (blue dashed line) staining in equine cadavers using the described ultrasound technique. a: pubis; b: sacrosciatic ligament; c: rectum; d: semimembranosus muscle; e: pelvic urethra; D: dorsal; V: ventral; Cr: cranial; Cd: caudal.

**Figure 4:**
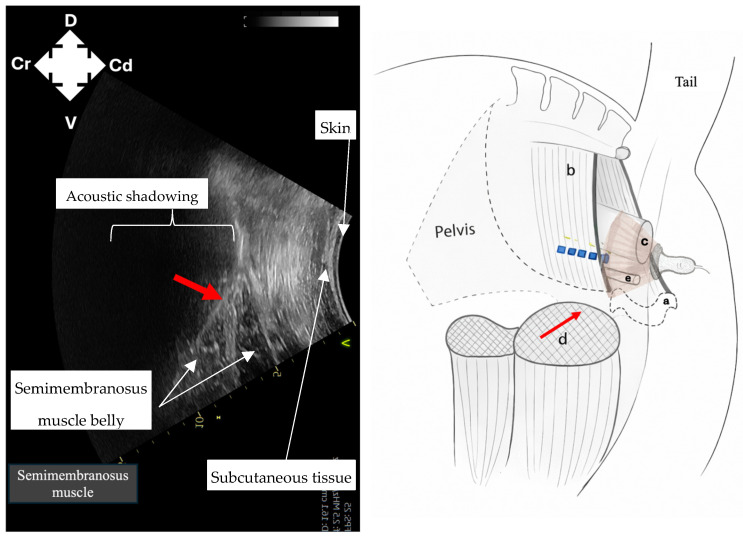
Ultrasound image and corresponding ultrasound probe placement to identify the semimembranosus muscle (red arrow) to guide needle placement for pudendal nerve (blue dashed line) staining in equine cadavers using the described ultrasound technique. a: pubis; b: sacrosciatic ligament; c: rectum; d: semimembranosus muscle; e: pelvic urethra; D: dorsal; V: ventral; Cr: cranial; Cd: caudal.

Identification of intrapelvic anatomy was performed using a curvilinear probe positioned laterally to the anus in a sagittal plane. Ultrasound machine depth was set to 15 cm and frequency set to 2–4 MHz. Gain was adjusted manually to optimise image contrast at deeper tissue levels. The pelvic brim (Figure 1) was identified first as a hyperechoic structure, with acoustic shadowing deep to it. Following this, dorsomedial redirection of the probe identified the rectum (Figure 2) as a rounded structure with wall layering and mixed echogenicity luminal contents. Ventrally, the pelvic urethra (Figure 3), round in cross-section, was identified. Subsequent lateral redirection enabled imaging of the semimembranosus muscle (Figure 4), including associated hyperechoic fascial planes. Any identification of vasculature was noted.

**Figure 5:**
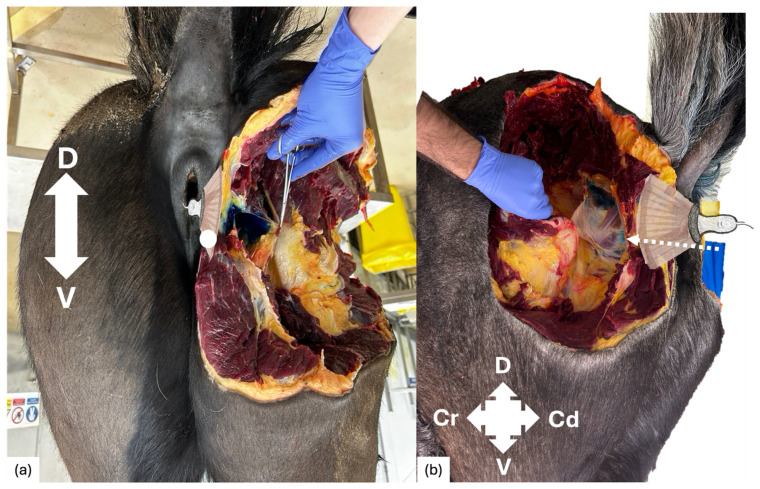
A caudal (**a**) and lateral (**b**) view of equine cadaver showing a successful injection attempt, with methylene blue dye deposited medial to the reflected sacrosciatic ligament. Needle (white dashed arrow) placement and sagittal ultrasound probe positioning are shown. D: dorsal; V: ventral; Cr: cranial; Cd: caudal.

Once the target area (bordered laterally by the semimembranosus muscle, medially by the rectal wall and ventrally by the pelvic brim) was identified, a 21-gauge 90 mm spinal needle was transcutaneously inserted, 1 cm ventral to and in plane with the ultrasound probe (Figure 1 and Figure 5). Under continuous ultrasound-guidance, the needle was advanced into the target area, which avoids the aforementioned structures. Once in position, methylene blue dye (15 mL) [12] was injected under ultrasound guidance into the target area. The needle was then withdrawn, and the entire process was repeated on the contralateral side.

Once bilateral dye injection had occurred, cadavers were transported to The Royal (Dick) School of Veterinary Studies’ Pathology Unit, sectioned at the level of the 4th lumbar vertebra and gross anatomic dissection of the pudendal nerve was performed within 24 h by the main researcher. When dissection was not performed immediately, cadavers were stored at 7 °C to maximise tissue preservation. Photographs of the dissection process were taken and the location and spread of injected dye were recorded. Pudendal nerve staining was considered successful if >2 cm of its length was stained [12]. Notes on the successful dissection approach were recorded.

Recorded data included: cadaver signalment; mass and BCS; time of euthanasia; cadaver position and time of USG injection; anatomic structures imaged and subjective image quality; depth of needle insertion; time of dissection; and extent of pudendal nerve staining. Other structures stained by dye misplacement or spread were recorded.

## 3. Results

### 3.1. Pilot Study (Two Fresh Equine Cadavers, One Standing Horse and One Fixed Equine Cadaver)

The first fresh cadaver, positioned in left lateral recumbency, was imaged using a linear ultrasound probe which produced a narrow field of view with loss of adjacent anatomical structures. Additionally, image resolution was poor in the far field of view. The subsequent use of a curvilinear probe produced a wider field of view with improved image resolution, enabling a more comprehensive assessment of the intrapelvic anatomy. Lateral positioning, high BCS (8/9) and poor perineal conformation (craniocaudal sloping of the vulva and a sunken anus) of this cadaver distorted the anatomy and affected image quality on the dependent side.

The second fresh cadaver, positioned in dorsal recumbency, was imaged using the technique described in the materials and methods which improved image quality compared to the laterally recumbent cadaver. Identification of the pelvic urethra, rectum, pelvic brim and semimembranosus musculature was possible. The pudendal nerve was not visible. The lower BCS (5/9) of the second cadaver with regular perineal conformation also facilitated imaging.

The third pilot imaging study, performed in a standing horse (BCS 5/9) using the ultrasound imaging technique described in the materials and methods, produced similar results to the dorsally recumbent cadaver with easy identification of the pelvic brim, rectum, pelvic urethra and semimembranosus muscle. Colour flow Doppler mode was used to confirm bilateral anechoic structures deep to the pelvic brim and lateral to the rectum as vasculature, likely corresponding to the internal pudendal artery.

**Figure 6:**
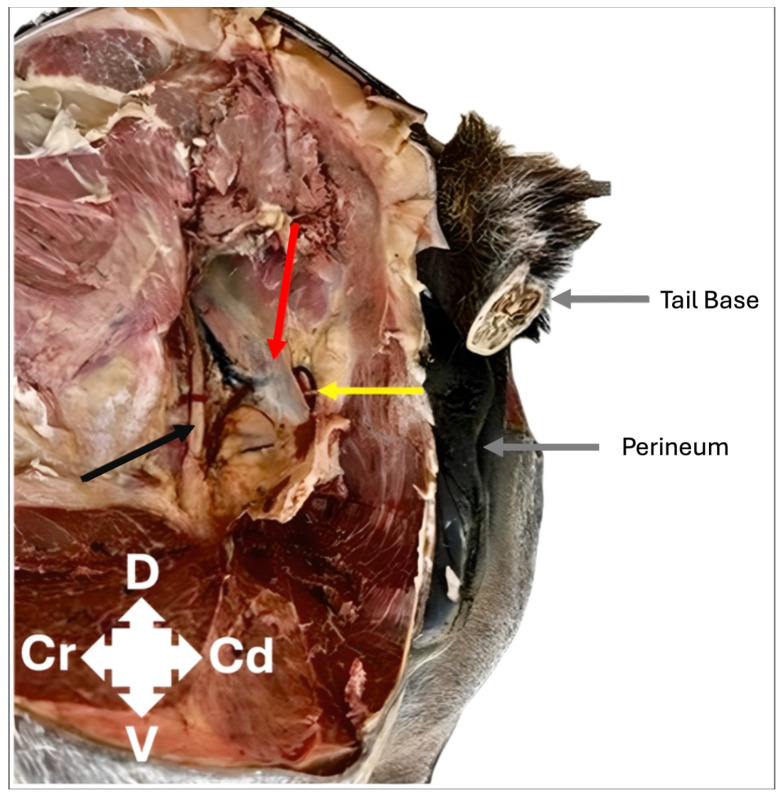
Lateral view of a fixed equine cadaver dissection identifying the sacrosciatic ligament (red), pudendal nerve (yellow) and sciatic nerve (black). D: dorsal; V: ventral; Cr: cranial; Cd: caudal.

Finally, a fixed equine cadaver specimen was dissected to identify the pudendal nerve and refine the dissection technique. A caudolateral dissection approach identified the sacrosciatic ligament separating the pudendal nerve (on the medial aspect) and the sciatic nerve (on the lateral aspect) (Figure 6).

The pilot phase produced the following eight findings: (i) dorsal recumbency is the optimal cadaver position; (ii) curvilinear probe use provides a wider field of view with superior image quality and improved resolution of deeper structures; (iii) optimal ultrasound probe positioning is ventrolateral to the anus; (iv) imaging of the pelvic brim, rectum, pelvic urethra and semimembranosus muscles can be reliably performed; (v) imaging of the pudendal nerve itself was not possible; (vi) the vasculature imaged in the standing horse may not be visible in recumbent cadaver specimens; (vii) higher BCS and poor perineal conformation may affect image quality; and finally (viii) the optimal dissection approach to the anatomical location of the pudendal nerve.

### 3.2. Main Study (Six Fresh Equine Cadavers)

Based on the findings of the pilot phase, all enrolled cadavers available during the study period were imaged, injected and dissected within 24 h of euthanasia. Six fresh cadavers were used, each allowing left and right-sided injection attempts (total 12 injections). One cadaver was imaged and injected in right lateral recumbency and five in dorsal recumbency.

The cadaver population of four geldings and two mares consisted of one Irish Sport Horse (cadaver 1), one Sport Horse (cadaver 2), one Clydesdale (cadaver 3), one Dales Pony crossbreed (cadaver 4), one Fell Pony (cadaver 5) and one Connemara (cadaver 6). Cadavers were numbered chronologically. The median cadaver body weight was 475 kg (360–550 kg) and median cadaver age was 16 years 7 months (13 months–20 years 7 months).

Imaging of intrapelvic anatomy (pelvic brim, pelvic urethra, rectum) and bilateral semimembranosus muscles was possible in all cadavers (Figure 1, Figure 2, Figure 3 and Figure 4). No vasculature was noted in any cadaver. Subjective assessment of image quality was recorded (Table 1). Ultrasonographic identification of the pudendal nerve per se was technically challenging and not possible in any cadaver.

Pudendal nerve staining was achieved in 5/12 injections (41.7%). Injections resulting in pudendal nerve staining occurred in cadavers 3 to 6. Staining occurred unilaterally in three cadavers (cadavers 3, 4 and 5) and bilaterally in one cadaver (cadaver 6). Staining failed bilaterally in two cadavers (cadavers 1 and 2). The average extent of pudendal nerve staining was 8.26 cm (7.1–8.9 cm) (Table 1). The average needle insertion depth was 87.5 mm (75–90 mm). When pudendal nerve staining was achieved, dye was widely distributed across the medial sacrosciatic ligament surface within a fascial plane (Figure 5). Misplacement of dye into pelvic limb musculature occurred in 1/12 injections, onto colon serosal surface in 4/12 injections and dye identification was not possible in 2/12 injections. Staining of the sciatic nerve did not occur in any cadaver, nor was dye present in the rectal muscularis, mucosa or lumen. Table 1 summarises the dye distribution on gross dissection.

The main study confirmed the main eight findings of the pilot study. Moreover, a staining rate of 41.7% was reported, with an increase in the staining rate from cadaver 3 onwards and an average extent of the pudendal nerve staining of 8.26 cm (7.1–8.9 cm).

## 4. Discussion

We report a transcutaneous USG approach for pudendal nerve blockade in horse cadavers. The pudendal nerve was stained in 41.7% of injections (cadavers 3 to 6) with methylene blue dye deposited into a fascial plane medial to the sacrosciatic ligament. Imaging of the pudendal nerve itself was not possible. Anatomical landmarks used to guide needle placement and dye deposition were the rectum, pelvic urethra, pelvic brim and semimembranosus musculature. While ultrasonography facilitated avoidance of critical intrapelvic structures, significant refinement of the described technique with further cadaver studies is necessary to improve staining success rate and reduce dye misplacement.

Incorporating the eight pilot phase learning points into the main study, ultrasound reliably identified critical intrapelvic structures, which were used as landmarks to guide needle advancement to the medial aspect of the sacrosciatic ligament. In general, the benefits of ultrasound guidance include: direct visualisation of anatomy; concurrent needle observation, which allows precise positioning and avoidance of critical structures; the assessment of injectate location and spread; and improved local block efficacy [22,23]. Due to the relatively small size and deep location of the equine pudendal nerve, we were unable to identify it using ultrasound as reported in cats, dogs and sheep [14,18,19]. Furthermore, the required depth of ultrasound penetration in large animals compromises image resolution, making pudendal nerve identification even more difficult. Increasingly in the veterinary and human literature, USG nerve block techniques utilising fascial planes have been described [24,25]. Conventionally, target nerves are identified using ultrasound and local anaesthetics are deposited in close proximity. However, the fascial plane approach instils a larger injectate volume which spreads and anaesthetises associated nerves. As the pudendal nerve follows the medial aspect of the sacrosciatic ligament in the horse [12] and identification of the pudendal nerve per se was not possible, a larger injectate volume deposited at this fascial plane, as described in this study, enabled staining of the pudendal nerve while sparing critical intrapelvic anatomy.

In our cadavers, dye misplacement into the semimembranosus muscle and onto the rectal serosa were the noted complications. Specifically, misplacement of dye into the semimembranosus muscle occurred in 1/12 injections and onto the rectal serosal surface in 4/12 injections; the clinical implication of this in vivo is not known. Interestingly, dye identification was not possible in 2/12 injections (cadaver 1). No inadvertent staining of the sciatic nerve or any other structure occurred. Reported complications of pudendal nerve blockade across several species and techniques include rectal and vaginal puncture, pudendal vessel puncture and injury to the operator when using a blind palpation technique [12,14,26]. Also, inadvertent staining or blockade of the sciatic nerve while targeting the pudendal nerve has been reported in a canine cadaver study [18]. However, that canine cadaver study used a transgluteal approach to the pudendal nerve, with a dorsomedial to ventrolateral needle trajectory. This necessitates needle placement across the sacrosciatic ligament. In contrast, we describe an in-plane approach to the pudendal nerve in equine cadavers, with a caudocranial needle trajectory medial to the sacrosciatic ligament. As the sacrosciatic ligament separates the pudendal (medial to) and sciatic (lateral to) nerves [12], the in-plane approach potentially reduces the risk of inadvertent sciatic staining by avoiding sacrosciatic ligament puncture.

With ultrasound guidance we were able to visualise the anatomy to a depth of 10–15 cm, though image resolution decreased with increasing depth. Direct visualisation enabled avoidance of critical intrapelvic anatomy during needle placement. For complete sensory blockade of the anogenital tract, bilateral blockade of the pudendal nerves or all three branches is required. The study by Gallacher et al. (2016) investigating equine pudendal nerve blockade with a peripheral nerve stimulator used external anal sphincter and perineal twitches to confirm needle placement [12]. The needle insertion depth reported by Gallacher et al. ranged 35–60 mm, compared to our injection depth of 75–90 mm. Injectate deposition guided by these twitches risks blockade of only the perineal and caudal rectal branches of the pudendal nerves, as seen in the anal twitch group in Gallacher et al. This could omit blockade of the third pudendal nerve branch, the dorsal nerve of the penis or clitoris, resulting in incomplete desensitisation of the anogenital region. Transrectal colour flow Doppler ultrasound guidance has been used experimentally in standing donkeys, providing a pudendal nerve success rate of 100% with a needle depth of 80 mm [16]. Transrectal ultrasound enables positioning of the ultrasound probe adjacent to the pudendal neurovascular bundle, producing higher image resolution at the needle’s target site. However, a transcutaneous approach avoids the complications associated with rectal examination and potential damage of previously mentioned structures via continuous visualisation of the needle’s path. The reduction in image resolution we experienced at deeper tissue levels, in addition to the lack of blood flow as a landmark in cadavers, could lead to needle tip misplacement and explain the lower success rate in this study. Future studies could assess the combined use of the described ultrasound technique with peripheral nerve stimulation; this method could allow avoidance of critical structures with ultrasound while providing additional confirmation of needle tip location with nerve stimulation.

An alternative technique for desensitisation of the equine perineum is the sacro- or inter-coccygeal epidural, commonly utilising one or a combination of local anaesthetics, opioids and alpha-2 agonists [27,28]. Although epidurals can be performed blindly with relative ease, complete sensory blockade is not guaranteed due to improper technique, anatomical abnormalities or membranes and adhesions within the epidural space [28]. Other complications associated with epidural techniques in horses include ataxia, sedation, recumbency and impaired recovery from general anaesthesia [28,29]. Successful pudendal nerve blockade preserves the advantage of anogenital desensitisation while eliminating the risks associated with epidural injections in horses.

We faced some expected challenges when performing the USG technique which may explain the pudendal nerve staining rate of 41.7%. These challenges included operator factors, namely the ergonomics of obtaining adequate images of anatomy and needle position, and patient factors which may have affected image acquisition and quality [30]. The operator in our study was not a trained imager but had prior training and cross-species experience in USG techniques [31]. In general, training and experience is key to the success of USG techniques [32]. The pudendal nerve staining in cadavers 3, 4 and 5 (unilateral success) and 6 (bilateral success) appears to demonstrate a learning curve; a larger number of cadavers in future studies would help to clarify this assumption. The quality of ultrasound images in this study varied, particularly during the pilot phase when lateral recumbency of a cadaver impeded complete identification of anatomy. Despite this, the first cadaver in the main study was imaged and injected in lateral recumbency due to the location of euthanasia and inability to support the cadaver in dorsal recumbency. This cadaver showed no pudendal nerve staining. All subsequent cadavers were imaged and injected in dorsal recumbency which improved visualisation of anatomy and needle position. In addition to body position, high BCS affected the image quality. The second cadaver with a body condition score of 7/9 had subjectively moderate image quality and unsuccessful attempts at pudendal nerve staining. All subsequent cadavers in dorsal recumbency with a low to ideal body condition score had either unilateral or bilateral pudendal nerve staining with the described technique. Although a limited number of cadavers were used, this may indicate that the body position and BCS are patient factors that contribute to staining success rate.

Our study has several limitations. First, the use of cadavers may affect the echogenicity of tissue planes used to guide needle placement, as well as the spread of injectate when compared to living tissues. As in other cadaveric studies, this should be considered when extrapolating conclusions to in vivo scenarios. Second, the ultrasonographic assessment of vasculature is not possible in cadavers and although no clear vascular laceration was noted on imaging or dissection, we cannot be certain this did not occur. Furthermore, blood vessels such as the internal pudendal artery could be used as an additional landmark for needle placement in vivo and could facilitate more accurate injectate deposition to the target area [16]. Indeed, imaging of the standing horse used during the pilot phase of this study allowed identification of vasculature lateral to the rectum and medial to the sacrosciatic ligament bilaterally, likely the internal pudendal artery. Assessing the location of these vessels in recumbent horses and their usefulness as an additional landmark for targeting the pudendal nerve, as reported in sheep, dogs and donkeys [16,17,19], should be evaluated in future studies. Third and finally, the imaging in this study was not performed by a trained imager which could have affected the ability to identify and recognise the target structure and other anatomical landmarks to guide needle placement. However, the inability to identify the pudendal nerve with ultrasound has been reported in other species. In retrospect, a blinded evaluator performing the nerve staining assessment should have been incorporated, as in similar studies [33].

## 5. Conclusions

We describe a transcutaneous USG injection of 15 mL of methylene blue dye, using landmarks of the rectum, pelvic urethra, pelvic brim and semimembranosus muscle, which enabled staining of the pudendal nerve in 41.7% of injections. The pudendal nerve was not imaged in any cadaver; however, alternative landmarks are suggested in line with our primary objective. Our results suggest that this technique, in its current form, cannot be recommended as a feasible technique without further refinement with subsequent cadaver studies, rejecting our secondary objective. After significant refinement and improvement in the nerve staining success rate, application of a transcutaneous technique in a clinical setting with local anaesthetic drugs could improve intra- and post-operative analgesia for horses undergoing procedures of the anogenital tract, while mitigating the risks associated with blind techniques. Future research should focus on: refining this technique in cadavers to improve the staining success rate; and assessing the feasibility of the technique in living horses.

## Figures and Tables

**Table 1:** Summary of cadaver population, subjective ultrasound imaging quality, methylene blue dye distribution, presence of staining and length of pudendal nerve staining with dye after USG targeting of the pudendal nerve in six equine cadavers. Pudendal nerve staining was considered successful when >2 cm of the nerve was stained.

Cadaver Number	Cadaver Details	Injection Side	Subjective Image Quality	Methylene Blue Dye Location	Length of Pudendal Nerve Staining	Staining/No Staining
**1**	Irish Sport Horse Gelding 16y0m Mass: ~500 kg BCS 6/9 Right lateral recumbency	Right	Poor	No dye identified.	0.0 cm	No staining
		Left	Poor	No dye identified.	0.0 cm	No staining
**2**	Sport Horse Mare 19y7m Mass: ~550 kg BCS 7/9 Dorsal recumbency	Right	Moderate	Semimembranosus muscle.	0.0 cm	No staining
		Left	Moderate	Serosal surface of rectum, focal staining of the apposed medial aspect of sacrosciatic ligament.	0.0 cm	No staining
**3**	Clydesdale Gelding 1y1m Mass: ~360 kg BCS 4/9 Dorsal recumbency	Right	Good	Medial aspect of sacrosciatic ligament and pudendal nerve with dye spread through fascial plane.	7.1 cm	Staining achieved
		Left	Good	Serosal surface of rectum.	0.0 cm	No staining
**4**	Dales Pony Cross Mare 16y1m Mass: ~500 kg BCS 4/9 Dorsal recumbency	Right	Good	Medial aspect of sacrosciatic ligament and pudendal nerve with dye spread through fascial plane.	8.9 cm	Staining achieved
		Left	Moderate	Scant amount of dye noted on serosal surface of rectum.	0.0 cm	No staining
**5**	Fell Pony Gelding 17y1m Mass: ~450 kg BCS 5/9 Dorsal recumbency	Right	Moderate	Serosal surface of rectum and right pelvic cavity.	0.0 cm	No staining
		Left	Good	Medial aspect of sacrosciatic ligament and pudendal nerve with dye spread through fascial plane.	8.7 cm	Staining achieved
**6**	Connemara Gelding 20y7m Mass: ~400 kg BCS 3/9 Dorsal recumbency	Right	Moderate-good	Medial aspect of sacrosciatic ligament and pudendal nerve with dye spread through fascial plane.	8.4 cm	Staining achieved
		Left	Moderate-good	Medial aspect of sacrosciatic ligament and pudendal nerve with dye spread through fascial plane.	8.2 cm	Staining achieved

## Data Availability

The original contributions presented in this study are included in the article/Supplementary Materials. Further inquiries can be directed to the corresponding author.

## Supplementary Materials

The following supporting information can be downloaded at: https://www.mdpi.com/article/10.3390/ani16060995/s1, File S1: Reporting Guidelines: CACTUS (Reporting *C*h*A*racteristics of *c*adaver *t*raining and s*u*rgical *s*tudies: The *CACTUS* guidelines) [21].

## Abbreviations

The following abbreviations are used in this manuscript:

USG	Ultrasound-guided
BCS	Body condition score
NSAID	Non-steroidal anti-inflammatory drug
ECVAA	European College of Veterinary Anaesthesia and Analgesia
