# Supplementary material for: Preliminary Investigation of a Transcutaneous Ultrasound-Guided Technique for Pudendal Nerve Block in Six Horse Cadavers
Source: Animals (Basel). 2026 Mar 23;16(6):995. doi: 10.3390/ani16060995 (PMC13024491; doi:10.3390/ani16060995)
Supplement: Supplementary file 1 [file animals-16-00995-s001.zip › animals-4175479-supplementary.pdf]

Supplementary S1.

Reporting Guidelines: CACTUS (Reporting *Ch**A*racteristics of *c*adaver *t*raining and s*U*rgical *s*tudies: The *CACTUS* guidelines) (Mantica et al. 2022).

The final version of CACTUS guidelines.

1. Approval of the use of the bodies with a clear statement of the institute that approves their use.
2. Number and gender of the bodies and/or organs used. If possible, report important clinical data such as BMI, basic medical history or previous non neglectable surgery.
3. State of conservation of corpses and/or parts of them, indicating the days of death before preservation.
4. In case of preparation and/or embalming of the body/organ, briefly indicate the methodology used (i.e. Fresh frozen, Thiel's technique, etc) and the time elapsed from the conservation procedure to the use of the corpse for training/study purposes.
5. Indicate the type of study for which they are used (anatomical study, surgical study, surgical training, device training, etc).
6. The type of fluids other than water with which the bodies come into contact during the study (i.e.Saline solution 0.9%, formaldehyde, etc) might be a useful additional but not mandatory information.
7. If cadaver specimens are sampled for pathological evaluation, the type and method of sampling might be a useful additional but not mandatory information.
8. Indicate the number and qualification of investigators/trainees actively involved in the cadaver study/training. Only people involved in organization and training should be present in the activities, since the use of human bodies for study should be treated with all the ethics they deserve.
9. Provide brief outcomes in terms of satisfaction in the use of the cadaver model through a short questionnaire to be administered to the trainees/investigators and comparing the different models used in the study (i.e. Thiel fixed cadavers vs fresh frozen; i.e. human cadaver model used vs another biological or non-biological model used). If the human cadaver is the only model used in the study provide an overall comment on satisfaction compared to that expected. When it is possible, also report objective data on the usefulness of the training model.

Mantica G, Leonardi R, Diaz R, Malinaric R, Parodi S, Tappero S, Paraboschi I, Álvarez-Maestro M, Yuen-Chun Teoh J, Garriboli M, Ortega Polledo LE, Soriero D, Pertile D, De Marchi D, Pini G, Rigatti L, Ghosh SK, Onigbinde OA, Tafuri A, Carrion DM, Nikles S, Antoni A, Fransvea P, Esperto F, Herbella FAM, Oxley da Rocha A, Vanaclocha V, Sánchez-Guillén L, Wainman B, Quiroga-Garza A, Fregatti P, Murelli F, Van der Merwe A, Gomez Rivas J and Terrone C (2022) Reporting ChAracteristics of cadaver training and sUrgical studies: The CACTUS guidelines. Int J Surg, 101:106619, doi:https://doi.org/10.1016/j.ijsu.2022.106619.
